# Habitat fragmentation impacts mobility in a common and widespread woodland butterfly: do sexes respond differently?

**DOI:** 10.1186/1472-6785-12-5

**Published:** 2012-04-27

**Authors:** Benjamin Bergerot, Thomas Merckx, Hans Van Dyck, Michel Baguette

**Affiliations:** 1 Muséum National d'Histoire Naturelle, CNRS-MNHN-UPMC, UMR 7204 CERSP, 55 Rue Buffon, Paris, 75005, France; 2 hepia Geneva, University of Applied Sciences Western Switzerland, Technology, Architecture and Landscape, Centre de Lullier, Route de Presinge 150, Jussy, CH-1254, Switzerland; 3 Theoretical Ecology and Biodiversity Change Lab, Centro de Biologia Ambiental, Faculdade de Ciências, Universidade de Lisboa, Campo Grande, Lisboa,, 1749-016, Portugal; 4 Biodiversity Research Centre, Earth and Life Institute, Université catholique de Louvain (UCL), Croix du Sud 4 bte, Louvain-la-Neuve, L7.07.04, B-1348, Belgium; 5 CNRS, USR 2936, Station d’ Ecologie Expérimentale du CNRS, Moulis, 09200, France

**Keywords:** Mean daily distances, Edge effect, Permeability, Dispersal cost, Dispersal benefit

## Abstract

**Background:**

Theory predicts a nonlinear response of dispersal evolution to habitat fragmentation. First, dispersal will be favoured in line with both decreasing area of habitat patches and increasing inter-patch distances. Next, once these inter-patch distances exceed a critical threshold, dispersal will be counter-selected, unless essential resources no longer co-occur in compact patches but are differently scattered; colonization of empty habitat patches or rescue of declining populations are then increasingly overruled by dispersal costs like mortality risks and loss of time and energy. However, to date, most empirical studies mainly document an increase of dispersal associated with habitat fragmentation. We analyzed dispersal kernels for males and females of the common, widespread woodland butterfly *Pararge aegeria* in highly fragmented landscape, and for males in landscapes that differed in their degree of habitat fragmentation.

**Results:**

The male and female probabilities of moving were considerably lower in the highly fragmented landscapes compared to the male probability of moving in fragmented agricultural and deciduous oak woodland landscapes. We also investigated whether, and to what extent, daily dispersal distance in the highly fragmented landscape was influenced by a set of landscape variables for both males and females, including distance to the nearest woodland, area of the nearest woodland, patch area and abundance of individuals in the patch. We found that daily movement distance decreased with increasing distance to the nearest woodland in both males and females. Daily distances flown by males were related to the area of the woodland capture site, whereas no such effect was observed for females.

**Conclusion:**

Overall, mobility was strongly reduced in the highly fragmented landscape, and varied considerably among landscapes with different spatial resource distributions. We interpret the results relative to different cost-benefit ratios of movements in fragmented landscapes.

## Background

Dispersal is a key feature in ecology, evolution and conservation biology [1,2]. It contributes to (meta-)population dynamics mainly via two processes: (i) population size regulation via density-dependent emigration, and (ii) (meta-)population persistence via (re)establishment and ˋtopping up´ of populations by dispersing individuals [3]. The probability that an individual will move between habitat patches, and the distance covered, will affect to what extent it will have different fitness opportunities and constraints [4]. The costs associated with movements across a landscape (e.g. energy expenditure, predation risk, risk of not finding suitable habitat resources) have important repercussions for the evolution of dispersal [5] and depend of the landscape complexity [6]. As a result, there is more intraspecific variation in dispersal characteristics (e.g. between populations that deal with different landscape-scale habitat configurations) than has been appreciated before [7,8]. Estimates of intra-specific variation are highly relevant when it comes to extrapolating movement parameters of a population in one landscape to another. Different landscape elements or biotopes are usually associated with different costs and benefits to a moving individual and this cost-benefit balance consequently varies among different landscapes [9].

In butterflies (and several other taxonomic groups), dispersal can be associated with different behavioural types of movements [10]. ‘Routine’ movements are associated with daily activities such as resource exploitation (e.g. foraging, mate-location); these movements are usually characterized by high levels of returning and loops [10]. The second type can be qualified as displacement movement and is characterized by fast and directed movements designed for considerable net displacement and settlement at some distance from the previous or natal site [10]. Hence, within a habitat patch, individuals are expected to adopt mainly explorative movement behaviour with frequent returns and loops, but they may sometimes switch to fast and directed movements (e.g. [11]). In ˋcontinuous´ landscapes with densely and regularly spread resources, routine movements are predicted to contribute more to net displacement than they would do in fragmented landscapes where resources are clustered in discrete patches at considerable distances relative to the scale of space-use by the average individual. Thus, the contribution of routine movements to dispersal will vary among (meta)populations and is expected to decline with an increasing degree of habitat fragmentation [12].

Counterbalancing the risk of local extinction, individuals are generally more mobile in landscapes where habitat resources are fragmented than in landscapes where such resources are more collocated, but only up to a certain threshold, above which mobility will be selected against [7,13,14]. The impact of habitat fragmentation on organisms varies according to intrinsic factors (e.g. flight ability), extrinsic factors (e.g. resource distribution across the landscape) and interaction effects between both factors [15]. Intrinsic factors have been shaped by evolutionary forces. Species of low but also intermediate mobility usually suffer the most from habitat fragmentation [16,17]. Although studies on common species do exist (e.g. the speckled wood butterfly [18-20], the Glanville fritillary butterfly, [21,22]), many studies on fragmentation processes focus on localized species [23,24] and most fragmentation studies are biased towards the lower end of the mobility spectrum. This typically includes rare species of conservation concern. By contrast, the common species have been largely overlooked even if fragmentation effects are also expected in common, widespread and even in species showing range expansion (by definition characterized by higher fragment occupancy and/or higher local abundance than rare species, [25]). As these are mainly species of intermediate mobility, it is expected that fragmentation will have even bigger effects than on sedentary species which may explain the current severe declines being observed for this group of organisms. Indeed, [17] showed that butterfly species with intermediate mobility were more likely to decline in abundance following habitat fragmentation than were butterflies with either high or low mobility. Even if they were less threatened than more scarce species, fragmentation processes could have a dramatic influence on genetic erosion of species considered as common [26].

Here, we address the relationship between mobility and habitat fragmentation in a common, widespread butterfly species, the Speckled Wood (*Pararge aegeria* L.). Several studies have considered the butterfly as relatively sedentary, but it is nevertheless a rapidly expanding species and, hence, we consider it such a species of intermediate mobility [27-29]. It occupies natural and semi-natural wooded biotopes, which are experiencing substantial alteration as a result of changes in land use [30]. In Western Europe it is typically well represented in such fragmented landscapes [31-33], although there is multiple evidence of phenotypic differentiation between populations from forested landscapes and populations from agricultural landscapes (e.g. [34-36]). For instance, butterflies of the latter landscape type were able to orient towards a wooded target habitat from twice as far than individuals of continuous woodland landscapes [14]. Moreover, previous studies on *P. aegeria* have shown changes in dispersal abilities between core and expanding populations toward the range margin in the UK [18,37]. Both habitat availability, and hence habitat fragmentation [27], as well as climate change [38] have an important effect on marginal rates of range expansion of *P. aegeria* populations and their dispersal abilities.

We report on the results of a mark-release-recapture (MRR) study on the movements of adult *P. aegeria* in a highly fragmented landscape for both males and females. We tested the hypothesis of higher probability to move in highly fragmented landscapes compared to less fragmented landscapes for males. Several empirical studies on *P. aegeria* have shown an increase, up to a certain degree, of dispersal movement with habitat fragmentation [39,40]. These studies refer to the ‘resource distribution’ hypothesis (i.e. butterflies in more fragmented landscapes have higher levels of mobility as resources are more scattered) [13,27]. For comparison, we re-analysed other MRR-datasets on males *P. aegeria* in differently fragmented landscapes. We compared the patterns using MRR datasets from three other landscape settings (detailed in [13] and [14]), and we analyze landscape-specific and population-specific variables to explain the observed patterns. In doing so, we were particularly interested in sexual differences in the most fragmented landscape. Even if several studies on dispersal in butterflies have pooled mark-release-recapture data of males and females (e.g. [41,42]), the behaviours and the costs of dispersal in fragmented systems differ between sexes [34,36,40]. Based on this sexual difference in flight ability in relation to habitat type, we tested potential differences in *P. aegeria* in three specific landscapes. We will discuss the impacts induced by high levels of habitat fragmentation on metapopulation functioning.

## Results

A total of 852 individuals were captured and marked in the Parc du Sausset (241 females and 611 males). 350 males and 69 females were recaptured (57.3% and 28.6%, respectively). Recapture events represented a total of 974 distances between two (re)capture-recapture points (838 for males and 136 for females). Recapture distances ranged from 0 to 1453 m and from 0 to 1618 m for males and females, respectively.

**Figure 1:**
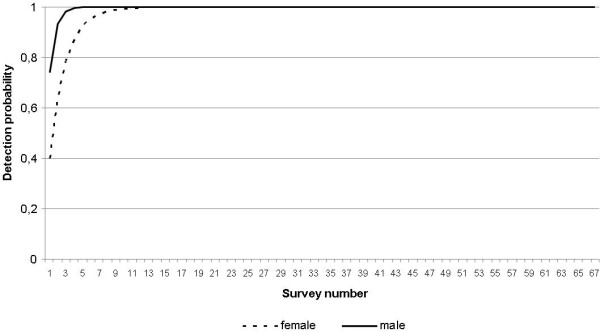
**Butterfly detection probabilities.** Relationship between detection probability rates of males (bold line) and females (dotted line) and the number of recapture surveys.

Estimation of detection probabilities in woodland patches within the urban park study area (Figure 1) showed that our sampling design with 67 surveys was sufficient to detect individuals in the eight woodland habitat patches with a detection probability close to 1. Three surveys were sufficient to reach a detection probability > 95% for males, whereas at least six were needed for females.

**Figure 2:**
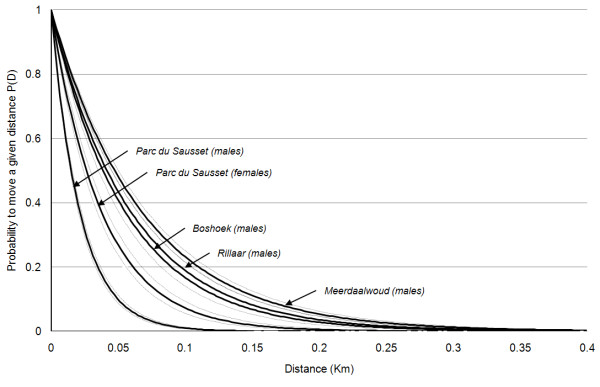
** Negative exponential dispersal kernels.** Sex-specific cumulative probabilities to move a given distance (km): P(D) (black bold line) ± SD (dotted lines) in the different landscape types.

**Figure 3:**
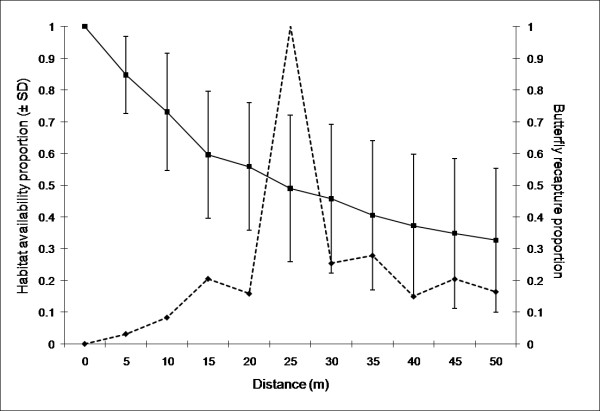
** Butterfly recapture distances.** Mean habitat availability proportion ± SD (black bold line) and butterfly recapture proportion (dotted black line) in relation to distance (meters) from individual capture locations.

The negative exponential distributions of distances in the urban fragmented landscape (Parc du Sausset) for males and females showed contrasting values of α (46.18 ± 2.50 and 26.26 ± 2.51, respectively; *P* < 0.001). Thus, males showed a much lower probability of moving long distances than did females (Figure 2). The average distance moved by individuals (1/α) was 21.6 m for males and 38.1 m for females. The recaptured proportion at a specific distance from capture sites showed that covered distances were not affected by the average amount of habitat patches available (Figure 3). In the urban fragmented landscape, α for males was significantly higher (Table 1) than α for males in landscapes dominated by deciduous oak woodland (Meerdaalwoud, α = 14.61 ± 0.77) and fragmented agricultural landscapes (Rillaar and Boshoek, α = 17.90 ± 1.26 and 16.63 ± 1.31, respectively). In the urban fragmented landscape, α for females was statistically larger than α for males in other landscapes (Table 1).

**Table 1:** butterfly α values

**Sites**	**Boshoek (males) (α = 16.63 ± 1.31)**	**Rillaar (males) (α = 17.90 ± 1.26)**	**Parc du Sausset (females) (α = 26.26 ± 2.51)**	**Parc du Sausset (males) (α = 46.18 ± 2.50)**
Meerdaalwoud (males)	t = −0.62	t = −1.38	t = −5.20	t = −4.93
(α = 14.61 ± 0.77)	*p = 0.54*	*p = 0.21*	*p < 0.001*	*p < 0.001*
Boshoek (males)		t = −1.99	t = −8.36	t = −7.85
		*p = 0.09*	*p < 0.001*	*p < 0.001*
Rillaar (males)			t = −6.42	t = −6.15
			*p < 0.001*	*p < 0.001*
Parc du Sausset (females)				t = −4.39
				*p < 0.001*

Comparison of α values between sites (α values for each site are mentioned between brackets, t: Statistic value of the *t*-test, p: p-value of the test).

In the urban fragmented landscape (Parc du Sausset), mean daily distances differed significantly (LR χ² = 8.41, *P* = 0.003) between males (30.6 m; 95% CI: 7.2 m) and females (47.0 m; 95% CI: 23.2 m). The best-fitting GLM showed that these daily distances were negatively related to the distance to the nearest woodland (LR χ² = 4.76, *P* = 0.03). Daily distances were negatively related to the area of the departure site, but only so for males (LR χ² = 4.98, *P* = 0.02), not for females (LR χ² = 0.76, P = 0.38).

## Discussion

We showed that *P. aegeria* individuals (both males and females) had a more sedentary daily movement pattern in a highly fragmented urban landscape compared to males in fragmented agricultural and more continuous woodland landscapes (data for females were not available in these two landscape types). We showed that movements of *P. aegeria* in fragmented urban areas corresponded to a very large α value, whereas we expected the opposite due to the high fragmentation level of the landscape, and hence, the scattered and heterogeneous distribution of habitat resources. More generally, we observed that daily distances were inversely related to the distance to the nearest woodland patch and they differed between sexes. Daily distances flown by males were related to the area of the woodland capture site, whereas no such effect was observed for females. Finally, we showed that habitat availability and sampling design had not biased results, on the one hand by constraining daily dispersal movements and on the other hand by leading to under-sampling one of the sexes.

Distances of daily movements of *P. aegeria* decreased with increasing distance to the nearest woodland habitat patch. Thus, inter-patch distances play a role for these movements and nearby landscape cues may facilitate moving across patches. Indeed, it has been shown that individuals of fragmented agricultural landscape populations are able to orient toward forested habitat, but only so from a distance of 100 m on average [14].

In *P. aegeria*, daily distance flown differs between males and females [40,43] in highly fragmented landscapes. Here, males, but not females, had higher probabilities to stay in large compared to small woodland patches. Indeed, male individuals are known to adopt territorial behaviour in sunspots on the forest floor [44,45]. But males may adopt one of two different mate-locating tactics: males actively search for females (i.e. patrolling) or wait for them in aggressively defended territories (i.e. territorial perching) [37,46,47]. These two strategies can generate high local abundances in woodland patches which may lead to density-dependent dispersal, as demonstrated for other butterfly species [48,49]. However, in our study, there was no evidence for a relationship between local patch abundance and movements. Our results only suggest that in large woodland areas, males show shorter daily movements. Two reasons may explain such a pattern: (1) it might be a consequence of decreasing patch boundaries with increasing patch area, or (2) males adopt perching behaviour more successfully in large woodland patches. We cannot neglect that these results may also be linked to the carrying capacity of woodland patches [48] and be influenced by higher woodland patch quality [33]. Indeed, landscape composition (i.e. quantity and quality of habitat resources present, e.g. [50]) and landscape configuration (i.e. spatial arrangement and connectivity of habitat resources) are key factors that influence dispersal processes and have a strong impact on local populations [3].

Compared to males, females had a higher probability of moving longer distances in our highly fragmented landscape, which may be due to the advantage of distributing eggs over a large area [51]. Moreover, single individuals of *P. aegeria* crossing open fields in Britain were all females, which also suggests that when females cross boundaries between woodland patches, they are more likely to continue their flight across the landscape than males [52]. Based on these results and by considering that males and females differ in the degree of long-distance dispersal, we expect the same differences in dispersal patterns between males and females to apply in fragmented agricultural and more continuous woodland landscapes. The low recapture percentage for females could be explained by two reasons. Firstly, females show more cryptic behaviour than males and secondly, females are thought to be much more significant for long-distance dispersal in *P. aegeria* than males [51]. Males have a larger propensity to return into a habitat patch in the butterfly *Speyeria idalia*[53]. In our study, there was no relationship between female movements and the variables related to the departure site or to landscape features. Hence, this suggests that males and females interact at different spatial scales with their environment. Due to their mating behaviour, males are strongly influenced by their immediate environment; more precisely, their ability to detect and pursue a female depends on the acuity of their eyes, the motion of the object, the background and the ambient level of illumination in the butterfly *Asterocampa leilia*[54]. Field studies on male behaviour in *P. aegeria* have frequently observed fast types of flight, with high levels of acceleration from a resting posture to passing objects as a typical component of the behavioural repertoire of territorial males [44,55]. Females will show this type of powerful, explosive flight much more rarely than do males (perhaps only to escape from predator attacks) and they have very different flight patterns altogether. They alternate between fluttering inspection flights above potential host grasses and dispersal flights that are regularly interrupted by basking stops [51,56]. Thus, at the landscape scale, females have a higher probability to move further compared to males. Female movements are mainly driven by mating and mediated by costs of the searching males (e.g. energy expenditure, time lost and enhanced predation risk, [57] and spreading their offspring).

However, even if females were better able to cover wider distances than males in the highly fragmented landscape, the high α values showed that average distances in the highly fragmented urbanised landscape were small for both males and females compared to α values for males in fragmented agricultural landscapes (Boshoek/Rillaar) and the landscape dominated by deciduous oak woodland (Meerdaalwoud). Indeed, compared to values of 31 other species reviewed in [15] and compared to values of male *P.aegeria* in fragmented agricultural landscapes (Rillaar and Boshoek) or in a woodland landscape (Meerdaalwoud), the parameter scaling the exponential negative distribution of dispersal distances showed high values. Compared to the value of 24.3 in the localized skipper butterfly *Hesperia comma*, [58], our study on *P. aegeria* females showed a similar α value (i.e. 26.3), whereas males had higher α values (i.e. 46.2). Such differences between sexes in dispersal kernels are rarely tested. Our results indicated that dispersal kernels should not be considered as species-specific, but rather as the results of the context- and condition-dependent dispersal process (e.g. [59]). Only British *Plebejus argus* showed a higher α value (126.6 [60]), but this is due to specificities concerning populations extremely isolated in habitat islands within the British landscapes [60]. Many factors may partly affect α values. The main bias occurred for males. Due to their behaviours (they can either defend a territory and adopt a waiting strategy, intercepting females passing through their territory, or instead may actively search for mates [47]), they have a very high probability of capture, and hence are usually over-represented in MRR datasets. This bias was not controlled for and may explain the large value of α measured, notably compared to the females.

In the context of dispersal modelling, recent studies have aimed to analyse dispersal kernels in various landscapes (e.g. for seeds [61,62]). Such models are currently used to study highly complex dispersal patterns. The evolution of dispersal kernels, which are themselves shaped by the environment, provides a valuable indication of selection acting upon species traits [63]. In this context, our study fits recent modelling studies where dispersal kernels emerged from movement rules [62,63]. More precisely, our study provides a novel extension to these recent modelling developments because our results show that individuals do not use a single species-specific fixed movement rule [62] but rather that sex-specific rules may apply too. These results are in agreement with other modelling studies [62,64,65].

Finally, the aim of this study was to assess dispersal evolution in relation to habitat fragmentation. Even if we showed here that individuals move within the landscape, and that males in fragmented habitats move less, we still have no idea whether they are ‘able’ to move more or whether the movement differences are due to some other factors than fragmentation (e.g. dispersal evolution).

## Conclusions

In this study, both males and females presented lower daily movements in urban fragmented landscapes contrary to results found in fragmented agricultural and in continuous woodland landscapes for males. The dispersal pattern observed here could be explained by the behavioural results of an outdoor cage experiment with *P. aegeria* butterflies that originated from different types of landscape [13]. They showed evidence for the "behaviour at boundaries" hypothesis (i.e. butterflies could have lower levels of mobility as they experience ‘hard’ habitat boundaries more frequently) rather than for the "resource distribution" hypothesis (i.e. butterflies in more fragmented landscapes would have higher levels of mobility as resources are more scattered). The effect was particularly significant in females [13]. Edge crossing behaviour is related to dispersal propensity and not necessarily to dispersal ability, and [13] argued that behavioural responses at habitat boundaries depend on the landscape type. In highly fragmented landscapes, because boundary crossings are characterised by a higher dispersal cost, butterflies could have lower levels of mobility [7,13,17,66]. In other words, daily movements are increasingly hindered by increasing fragmentation; they should increase in amplitude with fragmentation (to a certain threshold indeed) and then drop off dramatically. This hypothesis was supported by predictions of the effect of patch area on emigration rate according to fragmentation [67]. Indeed, butterflies were more likely to leave small patches than large ones in fragmented landscapes. Such a pattern where the majority of individuals remained within the larger patches is characteristic of local populations within a metapopulation system [58]. The case of *P. aegeria* in differently fragmented landscape systems provide an interesting scope for further research on the costs of dispersal and mobility in general [5].

## Methods

### Study species

The Speckled Wood (*Pararge aegeria* L.) is a satyrine butterfly using flight for almost all adult activities including mate location, foraging, host plant searching, oviposition and dispersal [68]. *P. aegeria* is a common species with a wide distribution throughout Europe [27]. This multivoltine species is mostly found in woodland biotopes, although it also occurs in fragmented, agricultural landscapes with hedgerows and small woodlots [13,69]. The larvae feed on various grass species [51].

### Study areas

**Figure 4:**
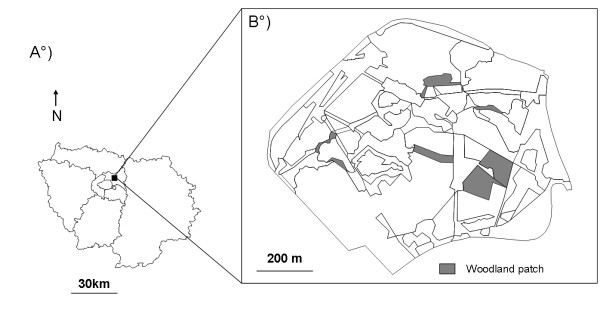
** Study sites.** A. Map of the Île-de-France region (Paris, France), and B. locations of the eight woodland patches sampled in the northern part of the Parc du Sausset.

We collected new Mark-Release-Recapture data (MRR) in an urban park in France (Parc du Sausset; 48°57'41.68"N, 2°30'35.28"E). The park (250 ha) is located in a dense urban matrix, surrounded by motorways. The study area consisted of a set of eight small woodland fragments (496–9625 m^2^) surrounded by a landscape with lawns and meadows (0.28 - 1.94 ha) in the northern part of the park (Figure 4). Woodland patches comprised a mixture of deciduous and coniferous tree species (i.e. *Quercus petraea, Quercus robur, Pinus sylvestris, Fagus sylvatica and Carpinus betulus*). Following the standardized nomenclature of the CORINE land cover classification (http://www.eea.europa.eu/themes/landuse/clc-download), biotopes were mapped into 44 classes.

We analysed and compared our data with movement data from the same species, obtained by the same MRR-protocol in 2000 in three study landscapes in Flanders (N-Belgium). One landscape was dominated by deciduous oak woodland (i.e. Meerdaalwoud, 1255 ha), whereas the other two were situated in fragmented agricultural landscapes (i.e. Rillaar and Boshoek, 361 ha (with ca. 6% habitat) and 757 ha (with ca. 11% habitat, respectively) (for further details, see [35,40]).

### Mark-release-recapture data

Following the method used in Belgium in fragmented agricultural landscapes (Rillaar/Boshoek) and the deciduous oak woodland landscape (Meerdaalwoud), we collected MRR data in France in the Parc du Sausset during 67 surveys (from 13 May to 2 October 2009) in eight woodland patches. Surveys were conducted only under favourable weather conditions (wind speed < 5 Bf, air temperature > 16°C, and > 75% sunshine, i.e. < 25% cloud cover). Similar to [35] in Belgium, we checked the whole area in each sampling site for males and females during each survey, and regularly changed the trajectory to avoid any spatial bias in recording. We captured butterflies using a hand net and marked them individually at first capture with unique numbers on the ventral side of the left hindwing with a fine, non-toxic, permanent marker (StaedlerLumocolor 313, Staedler, Nürnberg, Germany). For each capture and recapture event, we recorded time, exact position by GPS, sex and mark number. Butterflies were released at the spot of capture.

For each population of a landscape type along the fragmentation gradient (i.e. fragmented urban landscape: Parc du Sausset; fragmented agricultural landscape: Rillaar and Boshoek; and woodland landscape: Meerdaalwoud), we calculated the dispersal kernel as the inverse cumulative proportion of individuals moving certain distances. We calculated the dispersal kernels for males and females separately as the contrasting behaviour between males and females could lead to a higher dispersal kernel for females compared to males [70]. Dispersal kernels were fitted to a negative exponential function (SAS®, proc NLIN, *P* < 0.001): P(D) = βe^-αD^ where the probability to move a given distance P(D) is dependent on the distance (D) and the constants α and β (e.g. [15]). Metapopulation dynamics of butterflies are highly sensitive to the value of α in negative exponential dispersal kernels [15]. Large values of α correspond to a low probability of moving long distances.

As variation in local abundance within habitat patches may lead to density-dependent dispersal kernels [35,48,71], an estimate of the abundance (*A*) was taken into account and calculated in the urban fragmented landscape for each woodland patch and each successive survey as follows:

(1) $$A=\frac{\left(Mt_{1}+1)\times (Ct_{2}+1\right)}{\left(Mt_{2}+1\right)}$$

where *Mt*_*1*_ represents the number of marked individuals at survey 1, *Ct*_*2*_ represents the number of recaptured individuals at survey 2 and *Mt*_*2*_ the number of newly marked individuals at survey 2.

### Statistical analyses

In order to test whether our sampling design in the urban fragmented landscape (with 67 surveys) allowed detecting all butterflies, we estimated the probability of detecting *P. aegeria* individuals according to the model presented by [72] using PRESENCE v2.2 (developed by Jim Hines of the U.S. Geological Survey; http://www.mbr-pwrc.usgs.gov/software/presence.html). The model allows calculating detection probabilities < 1. Non-detection of an individual does not mean that the individual was absent from the sampled site if detection probability was < 1.

**Figure 5:**
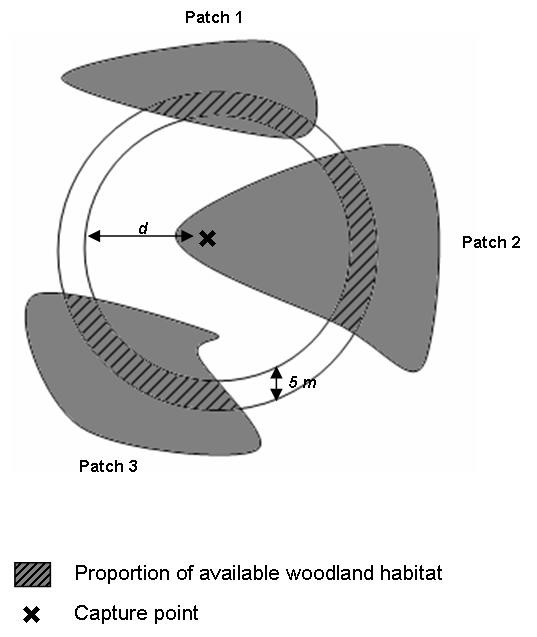
** Available woodland habitat calculation.** Proportion of available woodland for a butterfly at distance *d* of the capture point (d varied from 0 to 50 meters).

We also tested if dispersal patterns observed in the urban fragmented landscape were influenced by habitat availability. In order to do so, we calculated the proportion of available woodland habitat in concentric rings with 50 m radius for each capture event (i.e. around each capture location) (Figure 5). We then compared the proportions of available woodland habitat up to a radius of 50 m and the proportion of butterflies recaptured according to their daily distance flown weighted by ring area and transformed to vary between 0 and 1. We chose 50 m as a radius limit because this value corresponded to the average habitat target detection distance (i.e. perceptual range) in a woodland *P. aegeria* population [14].

As dispersal kernels were fitted to a negative exponential function P(D) = βe^-αD^, we used linear relationship ln(P(D)) = ln(β) – αD and Student statistics to compare α values between sites.

Daily distances (defined as the distance in meters covered by a butterfly in one day) were analyzed with General Linear Models (GLMs) using sex, distance to the nearest woodland, area of the nearest woodland, area of the departure site and abundance of individuals in the departure patch as explanatory variables. GLMs were constructed assuming normal distributions for the daily distances and a backward selection procedure of non-significant factors was used to select the best-fitting model (based on the Akaike Information Criterion, [73]). The full model included the daily distances as a dependent variable with all the explanatory variables mentioned above and all their two-way interactions. For the GLMs we used type III ANOVA with the associated P-values. All statistical analyses were performed with R2.10.1^©^.

## Competing interests

The authors declare that they have no competing interests

## Authors’ contributions

BB and MB conceived and designed the experiment. BB and TM carried out the field work. BB carried out data analysis. BB, TM, HVD and MB designed and helped to draft the manuscript. All authors read and approved the final manuscript.

## Authors' information

^1^Muséum National d'Histoire Naturelle, CNRS-MNHN-UPMC, UMR 7204 CERSP, 55 Rue Buffon, 75005 Paris, France. ^2^ hepia Geneva, University of Applied Sciences Western Switzerland, Technology, Architecture and Landscape, Centre de Lullier, route de Presinge 150, CH-1254 Jussy, Switzerland. ^3^ Theoretical Ecology and Biodiversity Change Lab, Centro de Biologia Ambiental, Faculdade de Ciências, Universidade de Lisboa, Campo Grande, 1749–016, Lisboa, Portugal. ^4^ Biodiversity Research Centre, Earth and Life Institute, Université catholique de Louvain (UCL), Croix du Sud 4 bte. L7.07.04, B-1348 Louvain-la-Neuve, Belgium. ^5^ CNRS, USR 2936, Station d’ Ecologie Expérimentale du CNRS, 09200 Moulis, France.

## References

[B1] Clobert J Danchin E Dhont AA, Nichols JD (Eds): Dispersal 2001 Oxford University Press, New York

[B2] Ricketts TH The matrix matters: Effective isolation in fragmented landscapes Am Nat 2001 158 87 99 10.1086/320863 18707317

[B3] Hanski I (Eds) The matrix matters: Effective isolation in fragmented landscapes Metapopulation ecology New York: Oxford University Press;

[B4] Spear SF Balkenhol N Fortin MJ McRae BH Scribner K Use of resistance surfaces for landscape genetic studies: considerations for parameterization and analysis Mol Ecol 2010 19 3576 3591 10.1111/j.1365-294X.2010.04657.x 20723064

[B5] Bonte D Van Dyck H Bullock JM Coulon A Delgado M Gibbs M Delgado M Lehouck V Matthysen E Mustin K Saastamoinen M Schtickzelle N. Stevens VM Vandewoestijne S Baguette M Barton K Benton TG Chaput-Bardy A Clobert J Dytham C Hovestadt T Meier CM Palmer SC Turlure C Travis JM Costs of dispersal Biol Rev 2012 in press 10.1111/j.1469-185X.2011.00201.x 21929715

[B6] Shreeve TG Dennis RLH Landscape scale conservation: resources, behaviour, the matrix and opportunities J Insect Conserv 2010 15 179 188

[B7] Schtickzelle N Mennechez G Baguette M Dispersal depression with habitat fragmentation in the bog fritillary butterfly Ecology 2006 87 1057 1065 10.1890/0012-9658(2006)87[1057:DDWHFI]2.0.CO;2 16676549

[B8] Stevens VM Pavoine S Baguette M Variation within and between closely related species uncovers high intra-specific variability in dispersal PLoS One 2010 5 e11123 10.1371/journal.pone.0011123 20559551 PMC2886073

[B9] Stevens VM Verkenne C Vandewoestijne S Wesselingh RA Baguette M Gene flow and functional connectivity in the natterjack toad Mol Ecol 2006 15 2333 2344 10.1111/j.1365-294X.2006.02936.x 16842409

[B10] Van Dyck H Baguette M Dispersal behaviour in fragmented landscapes: Routine or special movements? Basic Appl Ecol 2005 6 535 545 10.1016/j.baae.2005.03.005

[B11] Ohsaki N Comparative population studies of three Pieris butterflies, Pieris rapae, Pieris melete and Pieris napi, living in the same area .2. Utilization of patchy habitats by adults through migratory and non-migratory movements Res Popul Ecol 1980 22 163 183 10.1007/BF02513543

[B12] Baguette M Van Dyck H Landscape connectivity and animal behavior: functional grain as a key determinant for dispersal Landsc Ecol 2007 22 1117 1129 10.1007/s10980-007-9108-4

[B13] Merckx T Van Dyck H Karlsson B Leimar O The evolution of movements and behaviour at boundaries in different landscapes Proc R Soc B 2003 270 1815 1821 10.1098/rspb.2003.2459 PMC1691440 12964984

[B14] Merckx T Van Dyck H Habitat fragmentation affects habitat-finding ability of the speckled wood butterfly, Pararge aegeria L Anim Behav 2007 74 1029 1037 10.1016/j.anbehav.2006.12.020

[B15] Stevens VM Turlure C Baguette M A meta-analysis of dispersal in butterflies Biol Rev 2010 85 625 642 20055815 10.1111/j.1469-185X.2009.00119.x

[B16] Snep RPH Opdam PFM Baveco JM WallisDeVries MF Timmermans W Kwak RGM Kuypers V How peri-urban areas can strengthen animal populations within cities: A modeling approach Biol Conserv 2006 127 345 355 10.1016/j.biocon.2005.06.034

[B17] Thomas CD Dispersal and extinction in fragmented landscapes Proc R Soc B 2000 267 139 145 10.1098/rspb.2000.0978 PMC1690516 10687818

[B18] Hughes CL Hill JK Dytham C Evolutionary trade-offs between reproduction and dispersal in populations at expanding range boundaries Proc R Soc B 2003 270 147 150 10.1098/rspb.2002.2207 PMC1809962 14667365

[B19] Hughes CL Dytham C Hill JK Modelling and analysing evolution of dispersal in populations at expanding range boundaries Ecol Entomol 2007 32 437 445 10.1111/j.1365-2311.2007.00890.x

[B20] Van Dyck H Van Strien AJ Maes D Van Swaay CAM Declines in common, widespread butterflies in a landscape under intense human use Conserv Biol 2009 23 957 965 10.1111/j.1523-1739.2009.01175.x 19637406

[B21] Hanski I Heino M Metapopulation-level adaptation of insect host plant preference and extinction-colonization dynamics in heterogeneous landscapes Theor Popul Biol 2003 64 281 290 10.1016/S0040-5809(03)00093-5 14522169

[B22] Ovaskainen O Hanski I From individual behavior to metapopulation dynamics: Unifying the patchy population and classic metapopulation models Am Nat 2004 164 364 377 10.1086/423151 15478091

[B23] Rabasa SG Gutierrez D Escudero A Relative importance of host plant patch geometry and habitat quality on the patterns of occupancy, extinction and density of the monophagous butterfly Iolana iolas Oecologia 2008 156 491 503 10.1007/s00442-008-1008-z 18327616

[B24] Dover J Settele J The influences of landscape structure on butterfly distribution and movement: a review J Insect Conserv 2009 13 3 27 10.1007/s10841-008-9135-8

[B25] Gaston KJ Blackburn TM Greenwood JJD Gregory RD Quinn RM Lawton JH Abundance-occupancy relationships J Appl Ecol 2000 37 39 59 10.1046/j.1365-2664.2000.00485.x

[B26] Honnay O Jacquemyn H Susceptibility of common and rare plant species to the genetic consequences of habitat fragmentation Conserv Biol 2007 21 823 831 10.1111/j.1523-1739.2006.00646.x 17531059

[B27] Hill JK Collingham YC Thomas CD Blakeley DS Fox R Moss D Huntley B Impacts of landscape structure on butterfly range expansion Ecol Lett 2001 4 313 321 10.1046/j.1461-0248.2001.00222.x

[B28] Hill JK Thomas CD Fox R Telfer MG Willis SG Asher J Huntley B Responses of butterflies to twentieth century climate warming: implications for future ranges Proc R Soc B 2002 269 2163 2171 10.1098/rspb.2002.2134 PMC1691143 12396492

[B29] Vandewoestijne S Van Dyck H Population genetic differences along a latitudinal cline between original and recently colonized habitat in a butterfly PLoS One 2010 5 11 e13810 13810.11371/journal.pone.0013810 21072197 10.1371/journal.pone.0013810 PMC2972211

[B30] Bergerot B Fontaine B Renard M Cadi A Julliard R Preferences for exotic flowers do not promote urban life in butterflies Landsc Urban Plan 2010 96 98 107 10.1016/j.landurbplan.2010.02.007

[B31] Chardon JP Adriaensen F Matthysen E Incorporating landscape element into a connectivity measure: a case study for the Speckled wood butterfly (Pararge aegeria L.) Landsc Ecol 2003 18 561 573 10.1023/A:1026062530600

[B32] Shreeve TG Habitat selection, mate location, and microclimatic constraints on the activity of the speckled wood butterfly Pararge aegeria Oikos 1984 42 371 377 10.2307/3544407

[B33] Schweiger O Dormann CF Bailey D Frenzel M Occurrence pattern of Pararge aegeria (Lepidoptera : Nymphalidae) with respect to local habitat suitability, climate and landscape structure Landsc Ecol 2006 21 989 1001 10.1007/s10980-005-6057-7

[B34] Karlsson B Van Dyck H Does habitat fragmentation affect temperature-related life-history traits? A laboratory test with a woodland butterfly Proc R Soc B 2005 272 1257 1263 10.1098/rspb.2005.3074 PMC1564113 16024390

[B35] Merckx T Van Dyck H Mate location behaviour of the butterfly Pararge aegeria in woodland and fragmented landscapes Anim Behav 2005 70 411 416 10.1016/j.anbehav.2004.12.005

[B36] Gibbs M Van Dyck H Butterfly flight activity affects reproductive performance and longevity relative to landscape structure Oecologia 2010 163 341 350 10.1007/s00442-010-1613-5 20372930

[B37] Hill JK Hughes CL Dytham C Searle JB Genetic diversity in butterflies: interactive effects of habitat fragmentation and climate-driven range expansion Biol Lett 2006 2 152 154 10.1098/rsbl.2005.0401 17148351 PMC1617171

[B38] Parmesan C Ryrholm N Stefanescu C Hill JK Thomas CD Descimon H Huntleyk B Kaila L Kullberg J Tammaru T Tennent WJ Thomas JA Warren M Poleward shifts in geographical ranges of butterfly species associated with regional warming Nature 1999 399 579 583 10.1038/21181

[B39] Kemp DJ Wiklund C Van Dyck H Contest behaviour in the speckled wood butterfly (Pararge aegeria): seasonal phenotypic plasticity and the functional significance of flight performance Behav Ecol Sociobiol 2006 59 403 411 10.1007/s00265-005-0064-1

[B40] Merckx T Van Dyck H Landscape structure and phenotypic plasticity in flight morphology in the butterfly Pararge aegeria Oikos 2006 113 226 232 10.1111/j.2006.0030-1299.14501.x

[B41] Petit S Moilanen A Hanski I Baguette M Metapopulation dynamics of the bog fritillary butterfly: movements between habitat patches Oikos 2001 92 491 500 10.1034/j.1600-0706.2001.920310.x

[B42] Schultz CB Crone EE Edge-mediated dispersal behavior in a prairie butterfly Ecology 2001 82 1879 1892 10.1890/0012-9658(2001)082[1879:EMDBIA]2.0.CO;2

[B43] Berwaerts K Van Dyck H Aerts P Does flight morphology relate to flight performance? An experimental test with the butterfly Pararge aegeria Funct Ecol 2002 16 484 491 10.1046/j.1365-2435.2002.00650.x

[B44] Wickman PO Wiklund C Territorial defense and its seasonal decline in the speckled wood butterfly (Pararge aegeria) Anim Behav 1983 31 1206 1216 10.1016/S0003-3472(83)80027-X

[B45] Vande Velde L Turlure C Van Dyck H Body temperature and territory selection by males of the speckled wood butterfly (Pararge aegeria): what makes a forest sunlit patch a rendezvous site? Ecol Entomol 2011 36 161 169 10.1111/j.1365-2311.2010.01257.x

[B46] Bergman M Wiklund C Differences in mate location behaviours between residents and nonresidents in a territorial butterfly Anim Behav 2009 78 1161 1167 10.1016/j.anbehav.2009.08.003

[B47] Van Dyck H Matthysen E Thermoregulatory differences between phenotypes in the speckled wood butterfly: hot perchers and cold patrollers? Oecologia 1998 114 326 334 10.1007/s004420050454 28307775

[B48] Kuussaari M Nieminen M Hanski I An experimental study of migration in the Glanville fritillary butterfly Melitaea cinxia J Anim Ecol 1996 65 791 801 10.2307/5677

[B49] Mennechez G Petit S Schtickzelle N Baguette M Modelling mortality and dispersal: consequences of parameter generalisation on metapopulation dynamics Oikos 2004 106 243 252 10.1111/j.0030-1299.2004.12965.x

[B50] Wagner HH Wildi O Ewald KC Additive partitioning of plant species diversity in an agricultural mosaic landscape Landsc Ecol 2000 15 219 227 10.1023/A:1008114117913

[B51] Shreeve TG Egg-laying by the speckled wood butterfly (Pararge aegeria): the role of female behavior, host plant abundance and temperature Ecol Entomol 1986 11 229 236 10.1111/j.1365-2311.1986.tb00298.x

[B52] Baker RR Vane-Wrigth RI, Ackery PR The dilemma: when and how to go or to stay 1984 Academic, London 279 296

[B53] Ries L Debinski DM Butterflies responses to habitat edges in the highly fragmented prairies of Central Iowa J Anim Ecol 2001 70 840 852 10.1046/j.0021-8790.2001.00546.x

[B54] Rutowski RL Postural changes accompany perch location changes in male butterflies (Asterocampa leilia) engaged in visual mate searching Ethology 2000 106 453 466 10.1046/j.1439-0310.2000.00551.x

[B55] Van Dyck H Matthysen E Dhondt AA The effect of wing colour on male behavioural strategies in the speckled wood butterfly Anim Behav 1997 53 39 51 10.1006/anbe.1996.0276

[B56] Pellegroms B Van Dongen S Van Dyck H Lens L Larval food stress differentially affects flight morphology in male and female speckled woods (Pararge aegeria) Ecol Entomol 2009 34 387 393 10.1111/j.1365-2311.2009.01090.x

[B57] Gotthard K Nylin S Wiklund C Mating system evolution in response to search costs in the speckled wood butterfly, Pararge aegeria Behav Ecol Sociobiol 1999 45 424 429 10.1007/s002650050580

[B58] Hill JK Thomas CD Lewis OT Effects of habitat patch size and isolation on dispersal by Hesperia comma butterflies: Implications for metapopulation structure J Anim Ecol 1996 65 725 735 10.2307/5671

[B59] Clobert J Le Galliard JF Cote J Meylan S Massot M Informed dispersal, heterogeneity in animal dispersal syndromes and the dynamics of spatially structured populations Ecol Lett 2009 12 197 209 10.1111/j.1461-0248.2008.01267.x 19170731

[B60] Thomas CD Hanski I Hanski I, Gilpin M Butterfly metapopulations 1997 Academic, San Diego 359 386

[B61] Hovestadt T Messner S Poethke HJ Evolution of reduced dispersal mortality and ‘fat-tailed’ dispersal kernels in autocorrelated landscapes Proc R Soc Lond B 2001 268 385 391 10.1098/rspb.2000.1379 PMC1088618 11270435

[B62] Heinz SK Strand E Adaptive patch searching strategies in fragmented landscapes Evol Ecol 2006 20 113 130 10.1007/s10682-005-5378-y

[B63] Barton KA Phillips BL Morales JM Travis JMJ The evolution of an 'intelligent' dispersal strategy: biased, correlated random walks in patchy landscapes Oikos 2009 118 309 319 10.1111/j.1600-0706.2008.16936.x

[B64] Roff DA The evolution of flightlessness in insects Ecol Monogr 1990 60 389 421 10.2307/1943013

[B65] Langellotto GA Denno RF Benefits of dispersal in patchy environments: mate location by males of a wing-dimorphic insect Ecology 2001 82 1870 1878 10.1890/0012-9658(2001)082[1870:BODIPE]2.0.CO;2

[B66] Hill KJ Dytham C Hughes CL Fellowes MDE, Holloway GJ, Rolff J Evolutionary changes in expanding butterfly populations 2005 CABI publishing, Oxon 519 533

[B67] Schtickzelle N Baguette M Behavioural responses to habitat patch boundaries restrict dispersal and generate emigration-patch area relationships in fragmented landscapes J Anim Ecol 2003 72 533 545 10.1046/j.1365-2656.2003.00723.x 30893963

[B68] Berwaerts K Van Dyck H Take-off performance under optimal and suboptimal thermal conditions in the butterfly Pararge aegeria Oecologia 2004 141 536 545 15309609 10.1007/s00442-004-1661-9

[B69] Dover J Sparks T A review of the ecology of butterflies in British hedgerows J Environ Manage 2000 60 51 63 10.1006/jema.2000.0361

[B70] Berwaerts K Aerts P Van Dyck H On the sex-specific mechanisms of butterfly flight: flight performance relative to flight morphology, wing kinematics, and sex in Pararge aegeria Biol J Linn Soc 2006 89 675 687 10.1111/j.1095-8312.2006.00699.x

[B71] Baguette M Schtickzelle N Negative relationship between dispersal distance and demography in butterfly metapopulations Ecology 2006 87 648 654 10.1890/04-1631 16602294

[B72] MacKenzie DI Nichols JD Lachman GB Droege S Royle JA Langtimm CA Estimating site occupancy rates when detection probabilities are less than one Ecology 2002 83 2248 2255 10.1890/0012-9658(2002)083[2248:ESORWD]2.0.CO;2

[B73] Akaike H A new look at the statistical-model identification Curr Contents Eng Technol Appl Sci 1981 51 22-22

